# Exploiting Light Polarization for Deep HDR Imaging from a Single Exposure †

**DOI:** 10.3390/s23125370

**Published:** 2023-06-06

**Authors:** Mara Pistellato, Tehreem Fatima, Michael Wimmer

**Affiliations:** 1 Department of Environmental Sciences, Informatics and Statistics, Ca’ Foscari University of Venice, 155, Via Torino, 30170 Venice, Italy; tehreem.fatima@unive.it; 2 Institute of Visual Computing & Human-Centered Technology, TU Wien, Favoritenstr. 9-11/E193-02, 1040 Vienna, Austria; wimmer@cg.tuwien.ac.at

**Keywords:** PFA camera, deep learning, high dynamic range imaging, polarimetric imaging

## Abstract

In computational photography, high dynamic range (HDR) imaging refers to the family of techniques used to recover a wider range of intensity values compared to the limited range provided by standard sensors. Classical techniques consist of acquiring a scene-varying exposure to compensate for saturated and underexposed regions, followed by a non-linear compression of intensity values called tone mapping. Recently, there has been a growing interest in estimating HDR images from a single exposure. Some methods exploit data-driven models trained to estimate values outside the camera’s visible intensity levels. Others make use of polarimetric cameras to reconstruct HDR information without exposure bracketing. In this paper, we present a novel HDR reconstruction method that employs a single PFA (polarimetric filter array) camera with an additional external polarizer to increase the scene’s dynamic range across the acquired channels and to mimic different exposures. Our contribution consists of a pipeline that effectively combines standard HDR algorithms based on bracketing and data-driven solutions designed to work with polarimetric images. In this regard, we present a novel CNN (convolutional neural network) model that exploits the underlying mosaiced pattern of the PFA in combination with the external polarizer to estimate the original scene properties, and a second model designed to further improve the final tone mapping step. The combination of such techniques enables us to take advantage of the light attenuation given by the filters while producing an accurate reconstruction. We present an extensive experimental section in which we validate the proposed method on both synthetic and real-world datasets specifically acquired for the task. Quantitative and qualitative results show the effectiveness of the approach when compared to state-of-the-art methods. In particular, our technique exhibits a PSNR (peak signal-to-noise ratio) on the whole test set equal to 23 dB, which is 18% better with respect to the second-best alternative.

## 1. Introduction

Conventional cameras typically record intensity information with a limited range of possible detectable values. Such limitations are not negligible when acquiring scenes exhibiting high dynamic content, as conventional cameras fail to capture the full dynamic range. In such cases, the result will either be overly bright or excessively dark, leading to the loss of information in certain areas. These areas are commonly referred to as overexposed or underexposed regions. Such acquisitions result in so-called low dynamic range (LDR) images. High dynamic range (HDR) imaging aims to recover missing information in those problematic scene regions, thereby increasing the final dynamic range [1]. Some of the key areas where HDR is applicable include scene understanding [2], robotics [3], autonomous driving [4], medical imaging [5,6], agriculture [7], and spacecraft imaging [8].

The literature proposes many HDR imaging techniques, most of which rely on taking multiple shots of the same scene using multiple exposures and merging them together. These techniques usually suffer from global or local alignment issues: the former arises when the camera is moving between acquisitions, while the latter is due to moving subjects in the scene [9]. To overcome this issue, researchers have been trying to recover missing pixel information using just one input image: such techniques are called inverse tone mapping (ITM) [10]. Other works investigated HDR reconstruction from single or multiple LDR images, exploiting several deep learning approaches [11,12].

PFA cameras are also shown to be useful for HDR imaging [3,13,14,15]: these cameras can capture four images simultaneously with polarizers at different orientations. Since each linear polarizer reduces the observed intensity according to the degree of polarization of incoming light, the filter acts as an exposure time reduction and, thus, an equivalent exposure time can be computed to perform HDR reconstruction. In principle this works, but only when light is highly polarized, otherwise all four filters will essentially receive similar responses. Since the degree of polarization in a real-world scene is generally low, the usage of a plain PFA camera is limited. In [15], we proposed an HDR reconstruction method with a PFA stereo camera setup. In these settings, we used two PFA cameras, with only one of them equipped with an extra linear polarizer positioned outside the lenses. The rationale of adopting such a stereo setup is to use the external filter to make the incoming light highly polarized, and exploit the resulting intensity attenuation, while acquiring the original scene as-is with the regular PFA camera. This is required because the polarization state of the incoming light is needed to correctly compensate the external filter and, thus, compute the correct equivalent exposure time. Experimental results show that the idea of using an additional polarizer in such a setting produces better quality HDR compared to the single PFA camera approach, but at the cost of increased complexity.

**Figure 1:**
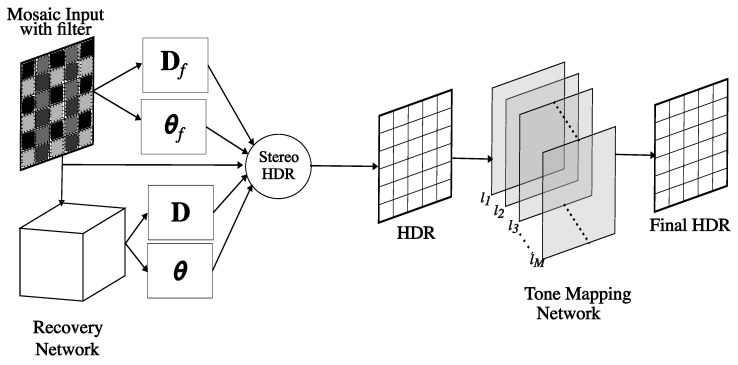
Our method’s pipeline overview. The mosaic input ($\hat{I}$) is the image taken from the camera with the linear polarizer mounted in front ($cam_{f}$) and is passed to the recovery network; this network simulates the response of the normal camera by predicting the actual angle (θ) and the degree (D) of polarization of the scene. $\theta_{f}$ and $D_{f}$ are the angle and degree captured by $cam_{f}$, computed from $\hat{I}$. HDR is reconstructed using polarization information and is further improved via the tone mapping network.

In this paper, we build on top of the method proposed in [15], with the goal of removing one camera and obtaining a simpler yet effective system to perform HDR reconstruction using one PFA camera. We propose a novel HDR reconstruction setup involving a single PFA camera with an additional linear polarizer outside the lenses, paired with a tailored data-driven model, which is trained to simulate the response of the missing camera (i.e., the one with no filter on). The proposed model estimates the scene AoLP (angle of linear polarization) and DoLP (degree of linear polarization) as if we have an actual second camera, and the resulting data are merged with the actual image with the additional filter. As a result, the proposed system performs HDR reconstruction from a single polarimetric image without the requirement of having a second camera. We can summarize the main contributions of this work as follows: we propose a novel data-driven approach that is able to effectively recover the original AoLP and DoLP of a scene from a single picture taken with an additional external polarizer. To our knowledge, this is the first work proposing such an “inverse problem” formulation for PFA cameras, exploiting a network architecture that takes into account the mosaiced pattern to characterize the acquired pictures. In this way, we obtain a virtual PFA stereo setup: the image coming from the physical camera and the network output can be merged together to compose an HDR picture, taking into account the equivalent exposure times associated with the scene features. Finally, a refinement step takes the computed HDR image and performs tone mapping via a specifically designed CNN architecture. The proposed reconstruction approach is shown in Figure 1. An extensive experimental study supports the effectiveness of the proposed method, including a comparison with state-of-the-art techniques. The rest of the paper is organized as follows: Section 2 describes the most relevant works connected to our method, then Section 3 briefly summarizes polarimetric basics and the stereo HDR reconstruction presented in [15]. The proposed technique is then presented in detail in Section 4, and finally both qualitative and quantitative experimental results are discussed in Section 5.

## 2. Related Work

### 2.1. Multiple LDR to HDR

The most common approach for HDR imaging is to take multiple images from the same scene with increasing exposure, which is usually referred to as “multi-exposure images”. The camera response function (CRF) is estimated using these images, and the inverse of CRF is applied to linearize the multi-exposure images. These images are aligned and merged together to produce the final HDR image. The following are state-of-the-art approaches designed to create HDR using multiple exposures.

#### 2.1.1. Classical Approaches

Numerous examples can be found in the literature, where researchers reconstructed HDR using multi-exposure images [16,17,18,19,20,21,22,23,24]. One of the earliest efforts in creating HDR images using multiple exposures was made by [16]; the authors introduced a novel method to recover the CRF as well as an HDR radiance map using multi-exposure images. Similarly, [17] proposed an HDR reconstruction method for handheld cameras since images taken by hand are more prone to artifacts, such as blurs. Images are first registered using the MTB algorithm, and then maximum likelihood is used to find the blur kernel and CRF. Mobile cameras have small sensor pixels, which means they can gather a very small number of photons per pixel. Due to this fact, images captured by mobile cameras have more noise and offer a very limited dynamic range. In [18], the authors introduced a rather unconventional way to take multiple LDR images with automatically adjusted exposures. This idea looks promising but it brings many problems, for example, when selecting auto-exposure for different real-world scenes, many hand-crafted features are used, which does not seem feasible, and it is an ’overhead’ to store such information. This pipeline is also time-consuming and may fail to produce desirable results in extreme cases (underexposed/overexposed).

Exposure fusion is an alternative approach to multi-exposure HDR imaging. Exposure fusion was introduced to simplify the pipeline for HDR imaging. This technique does not require CRF estimation and does not produce an intermediate HDR image, thus eliminating the tone mapping step. Although this technique does not provide an increase in the dynamic range, it produces visually pleasing results. Exposure fusion works well for static scenes while it suffers from artifacts in the case of moving objects or a shaking camera. Reference [1] proposed such a method in which multi-exposure images are merged in multi-resolution fashion on the basis of simple measures, such as saturation, well-exposedness, and contrast. The results produced by different measures look visually different, and each introduces its own artifacts.

#### 2.1.2. Learning-Based Techniques

Nowadays, learning-based techniques are employed in a variety of applications, being able to solve even complex problems [25,26,27]. Researchers have also investigated the creation of HDR using multiple LDR images by designing deep learning methods. To fuse a set of multiple exposed images together and generate an artifact-free HDR image, [28] designed the first learning-based approach. This is a CNN that takes two images (underexposed and overexposed) as input, with three components: in the first component low-level features are extracted, then these features are fused in the second layer. Finally, a reconstruction component generates the output HDR image. In [29], the use of GANs for HDR generation is proposed for the first time. The generator is formed by three parts: self-attention, local details, and merge block. The discriminator has a rather simple architecture and tries to discriminate between ground truth and generated HDR in terms of probability (0…1). Reference [30] proposed a CNN-based approach to generate ghost-free HDR for dynamic scenes. They aligned three images of the dynamic scene taken with increasing exposure using a classical optical flow algorithm. These aligned images were later merged using the CNN-based approach to produce HDR. Instead of an end-to-end mapping, [31] splits the HDR creation from dynamic input images [30] into two sequential tasks. The first is an encoder–decoder named AlignNet, which aligns three images with low, mid, and high exposures. These exposures are merged together by the next CNN called MergeNet, and the final HDR image is estimated. References [32,33] are also specifically designed to estimate HDR outputs for dynamic scenes and tested on the Kalantari [30] dataset. A self-supervised method is proposed in [34], where a set of three bracketed–exposed LDR images was used to create HDR patches for self-supervision based on static and well-exposed areas of the image. FlexHDR [35] takes an arbitrary number of LDR images as input and computes the optical flow between these differently exposed images using a flow network. After flow estimation, the FlexHDR model addresses uncertainties caused by exposure and alignment via an attention network, and the final HDR is generated by a multi-stage fusion-based merging network. Other learning-based methods can be found at [36,37,38,39,40,41,42,43,44,45].

### 2.2. Single LDR to HDR

The above-mentioned classical and learning-based techniques work well when the camera is mounted on a tripod and the scene is static, but if this is not the case, the output could suffer from the following problems. First, it may produce global (ghost-like) artifacts due to camera motion, and local artifacts can be caused by the movement of objects captured in the scene. Second, multiple LDR and HDR techniques cannot be applied to the existing single LDR image legacy content. To overcome the issues posed by multi-exposure methods, *inverse tone mapping* (ITM) is introduced with the aim of reconstructing the HDR image from a single exposure. Alignment and merging steps are no longer required while the HDR radiance map is reconstructed directly from the input image. These techniques simplify the HDR reconstruction pipeline and are useful for converting legacy data from the LDR domain to HDR. However, reconstructing HDR through a single exposure is an ill-posed problem and ITM techniques are underperforming when compared to exposure bracketing.

#### 2.2.1. Classical

A huge amount of image and video data is produced for visualization on outdated display devices. The widespread adoption of HDR displays has created a need for converting this historical data from LDR to HDR and making it compatible with these new devices. In this case, traditional HDR image generation algorithms fail, hence, the idea of ITM was introduced by researchers to enhance LDR content in order to display it on HDR devices. In [46], the authors proposed a support vector machine (SVM)-based approach for both images and videos. In the proposed solution, the ITM operator entirely depends on the decision of the SVM, and in some cases, the SVM fails to identify the scene type, which can result in erroneous decisions, eventually yielding unexpected results in the final HDR. Similar ITM methods [10,47,48] require parameter tuning and manual input; for this reason, in such cases, it is not easy to apply these methods because they could lead to the unrealistic output of low-quality HDR images. In general, conventional ITM methods have limited performance because the information is lost in the saturated and underexposed areas of the images, which makes the recovery of the correct CRF from a single image nearly impossible. As a result of this information loss, ITM-based methods produce HDR images with limited quality.

#### 2.2.2. Learning Based

Learning-based ITM HDRCNN proposed in [49] is a CNN autoencoder aimed at improving saturated areas of arbitrary exposed images. One limitation of the proposed technique is that it fails in the case of underexposed regions, and may introduce artifacts if large overexposed areas are present in the image. Reference [50] designed an encoder–decoder network, which takes the LDR image of the random exposure as input, and instead of directly producing the HDR image, similar to other ITM methods, it generates bracketed images with changing exposures. Due to the unavailability of appropriate datasets for training, the authors synthesized multi-exposure LDR images from existing datasets. Their technique can introduce artifacts with real-life data, especially in the case of highly overexposed regions. ITM-net [51] is another CNN proposing an ITM method, which takes an image in the LDR domain and predicts the tone-mapped HDR image. The LDR and HDR domain images are different, but in ITM-net, the authors did not take into account this fact and did not perform the domain transfer. ExpandNet [11] is an end-to-end CNN ITM method that consists of three branches: local, dilation, and global; each one targets a different level of information in the image. The outputs from the three branches are merged at the end and the final HDR is generated. ExpandNet seems to work well to recover the overexposed areas but fails to recover missing information in the dark regions of the image and, overall, produces darker results. HSVNet [52] is a U-Net-like model that converts arbitrarily exposed LDR images to HDR images. The proposed model works in the HSV (hue saturation value) color domain and introduces custom loss with masks computed over S and V channels to differentiate between underexposed and overexposed areas. Reference [53] suggests using a weakly supervised network that essentially recovers CRF using a single input image, which can generate any number of bracketed exposure images. This method avoids the alignment and merging steps of the classical multi-exposure HDR pipeline, but could introduce hallucination artifacts in some challenging regions of the image. More deep learning-based example methods include [12,54,55,56,57].

### 2.3. Novel Sensors for HDR

Spatially varying exposure (SVE) [58] is a specialized camera sensor, which is designed to simultaneously capture multiple exposures. The works [59,60] are based on a similar idea as SVE [58] but with different sensor pattern arrangements. These aforementioned methods overcome the issue of image alignment but they all suffer from spatial resolution reduction. There are cameras specifically designed to capture HDR content, for instance, Arri Alexa, Sony F65, and Omron [61]. SpheronVR and PanoScan cameras are designed to capture HDR panoramas [10], although these HDR cameras produce high-quality content, they are still very expensive.

### 2.4. PFA Camera-Based HDR

The work presented in [3] made the first effort to create single-shot HDR images using PFA cameras. The authors treated the four channels as images taken with different exposures, and the final HDR was reconstructed using the existing method [16]. An extension of this work was proposed in [13], where they adopted an 18-level autoencoder architecture similar to the one introduced in [50]. The major drawback of these approaches is that real-world scenes rarely exhibit high DoLP, and this is required to correctly map a polarimetric image into an image with equivalent exposure time. To mitigate this issue, the authors proposed another technique, named DPHR [14]. In order to improve poorly exposed pixels with low DoLP, [14] generated HDR with a PFA camera by using the degree of polarization as the cue in a CNN (similar to U-Net [62]) to create a feature mask. Image areas with high DoLP were reconstructed using a traditional approach (as in [3]), while pixels with moderate DoLP were reconstructed with both CNN and traditional techniques. Similar to the aforementioned single LDR to HDR techniques, PFA-based HDR methods also encounter the challenge of poor performance in underexposed areas. In [15], we introduced a novel stereo PFA camera setup, which provides a significant increase in the dynamic range of the HDR image, compared to using single PFA cameras. The HDR images computed with [15] are visually more pleasing and closer to the ground truth when compared to other existing methods. Despite these benefits, the stereo setup involves certain constraints, including being expensive, being complicated to manage, and image alignment issues. In Section 3, we introduce the basic concepts and equations for PFA-based HDR reconstruction, provide more details about the stereo setup and the model we employed to compute the final reconstruction with two cameras, and discuss the mentioned drawbacks.

## 3. HDR with Stereo PFA Cameras

Polarization is a fundamental property of light that arises from its vector nature [63]. When captured, this feature can be exploited for several applications, such as quality control [64], material classification [65], 3D reconstruction [66,67,68], and remote sensing [69,70], to name a few. The method used to acquire the polarization state of a scene often consists of placing a linear polarizer in front of an ordinary camera and rotating it to take pictures with different angles. Considering a single pixel, the observed intensities can be used to compute the Stokes vector $S=(S_{0},S_{1},S_{2},S_{3})$, which is a mathematical structure representing the polarization state of a light beam. From the values in *S*, we can easily compute the AoLP (angle of linear polarization) and DoLP (degree of linear polarization) as follows:

(1) $$\mathrm{DoLP}=\frac{\sqrt{S_{1}^{2}+S_{2}^{2}}}{S_{0}},\;\;\;\mathrm{AoLP}=\frac{1}{2}\arctan\frac{S_{2}}{S_{1}}.$$

Note that $S_{3}$ corresponds to circular polarization, which cannot be observed without the use of a retarder, which is, in general, rarely observable in nature [71]. As in multi-shot HDR, the described setup with a rotating polarizer brings several disadvantages related to the need for image alignment and filter calibration. To avoid such problems, polarimetric filter array (PFA) cameras have been introduced. This device mounts an internal filter that allows taking polarized images with four pre-defined filter orientations ($0^{\circ },45^{\circ },90^{\circ }$, and $135^{\circ }$) in a single shot.

**Figure 2:**
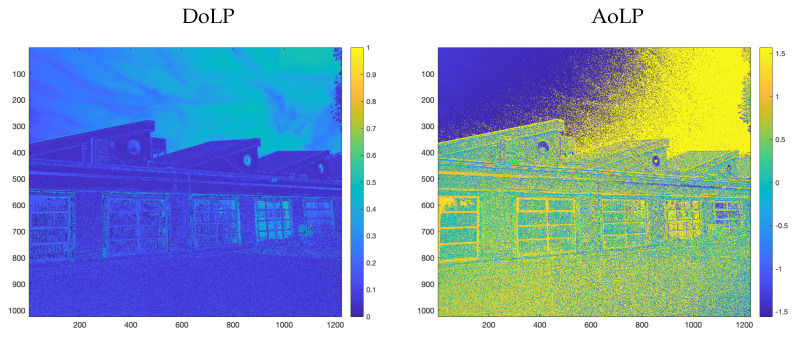
An outdoor high dynamic scene. The left image is the DoLP∈[0,1] and the right is the $\mathrm{AoLP}\in [-\frac{\pi }{2},\frac{\pi }{2}]$, recorded with the PFA camera.

If we focus on a single camera pixel, a polarizer filter with an angle α reduces the incoming image irradiance $\hat{I}$, according to the following equation, depending on the incoming light polarization state:

(2) $${\hat{I}}^{\prime }=\frac{1}{2}\hat{I}\left(1+D\cos(2\theta -2\alpha )\right)$$

where D∈[0,1] is the incoming DoLP for that pixel and θ∈[−π,π] is the AoLP for the same pixel, while α∈[−π,π] is the orientation of the linear polarizer with respect to the camera’s x-axis. In the case of PFA cameras, we observe macro-pixels, where each macro-pixel consists of four channels corresponding to four different filter orientations, meaning that α is going to assume fixed values ($0^{\circ },45^{\circ },90^{\circ }$, or $135^{\circ }$), as mentioned. As a consequence of this attenuation, the usage of such filters can be related to a reduced shutter speed and, thus, exploited to perform HDR reconstruction from the four images. However, Equation (2) clearly shows that the amount of attenuation is proportional to the DoLP D. In general, high DoLP is seldom observed in a general scene, as shown in Figure 2, where the DoLP and AoLP of an outdoor scene are displayed. Indeed, the DoLP image shows quite low values, except for the sky region, which is partially polarized with respect to the rest of the scene.

**Figure 3:**
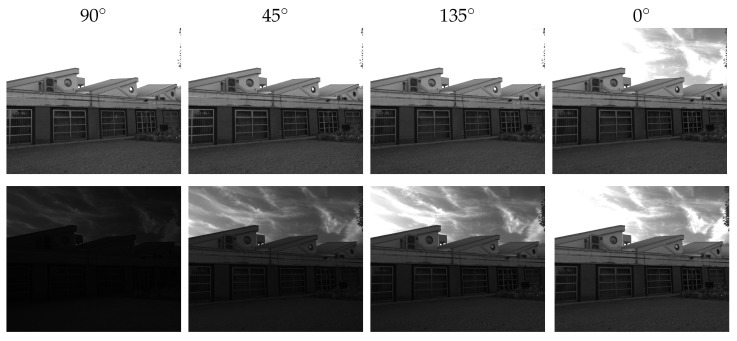
Images taken with the stereo camera setup introduced in [15]. The top row refers to demosaiced polarized filter images taken with a PFA camera: all channels essentially look the same. The bottom row shows the same scene taken with the second PFA camera and external linear polarizer; it produces images similar to exposure bracketing.

The top row of Figure 3 shows the same scene captured by the four channels of a PFA camera (filters oriented at $0^{\circ },45^{\circ },90^{\circ }$, and $135^{\circ }$). Since there is some polarized light coming from the sky, the intensity in this area exhibits a different response across the different filters, but only in one image. Since the rest of the scene has DoLP ≈0, there are no variations and, thus, in this area, HDR reconstruction would be useless. Fatima et al. [15] proposed a solution to overcome this issue by mounting an additional external linear polarizer on the PFA camera, so that incoming light is polarized ideally with DoLP ≈1. Figure 3 (bottom row) displays the same scene captured with such an additional linear filter in front of the lenses. This time, the response observed in the four channels is similar to what would be obtained through exposure bracketing. Since the external filter overrides the polarization state of the original scene (needed to recover the equivalent exposure time), in [15], a stereo setup is proposed, such that a second PFA is used to acquire the original image (i.e., the AoLP and DoLP). This camera setup results in a model through which the per-pixel exposure time can be effectively estimated.

Suppose we observe a scene with irradiance I, AoLP θ, and DoLP D. Employing a regular PFA camera to capture the scene, according to Equation (2), we obtain four different intensity images $I_{0},I_{45},I_{90},I_{135}$, one for each internal polarizer orientation $\alpha_{i}$, where $i\in \{0^{\circ },45^{\circ },90^{\circ },135^{\circ }\}$. Note that θ and D are intrinsic features of the scene since they depend on the polarization state of the acquired light, and can be computed from the four $I_{i}$. When the same scene is captured by another PFA camera, denoted as $cam_{f}$, with an additional externally mounted linear polarizer at an angle $\alpha_{f}$, we obtain different AoLP and DoLP, denoted, respectively, as $\theta_{f}$ and $D_{f}$. The combination of the external and internal filters can be modeled by applying the relationship in Equation (2), producing the following irradiance for the *i*-th filter:

(3) $${\hat{I}}_{i}=\frac{1}{4}I\left(1+D\cos(2\theta -2\alpha_{f})\right)\left(1+D_{f}\cos\left(2\theta_{f}-2\alpha_{i}\right)\right).$$

This relation is exploited to compute equivalent exposure times for each pixel and proceed with HDR reconstruction. Experiments from the original paper show that by merging the responses of both cameras, the dynamic range of images is significantly increased as compared to using a single PFA camera. Despite these benefits, the described setup has some limitations:

1. It requires two PFA cameras mounted on a rigid structure, which is expensive.
2. The stereo setup is complicated to manage and not easy to use (e.g., shutter synchronization or the calibration of the camera’s response function).
3. Although two cameras have a minimum baseline, there is still a need to align the responses of both cameras, and mapping can be erroneous, especially for objects near the cameras.

## 4. Single-Shot HDR with the PFA Camera

The mentioned issues can be resolved by reducing the stereo’s camera setup to a single physical camera with an external filter; simulating the response of the other camera to obtain the actual AoLP and DoLP of the scene can be obtained to proceed with the HDR reconstruction, as described in the previous section. In this work, we present a technique that effectively allows discarding the second camera, thus reducing the disadvantages of the previous stereo configuration.

The idea is to acquire the scene, employing only one PFA camera with the additional linear polarizer, and then using a data-driven model to simulate the outcome of an unfiltered PFA camera based on the captured image. To this end, we trained a neural network to predict the actual AoLP and DoLP of a scene from a filtered image, observing that (i) some information is still preserved after passing through the linear filter (which is not perfect) and (ii) the training process demonstrates the network’s ability to generalize to typical configurations, to devise the expected values for the angle and degree. In this way, we are able to create a virtual stereo camera setup composed of the real camera and the output of the discussed model. Then, these two images can be combined, as already discussed in the previous section, and presented in [15]. Note that the computed HDR image exhibits a broader range of values compared to those captured by a regular camera; indeed, we obtain real values that should typically be quantized to 256 levels to visualize the results. Usually, this step involves a tone mapping technique; therefore, we also propose a second neural model that is designed to take the computed HDR image and compute the tone-mapped version, which improves the final output. Figure 1 presents an overview of the complete pipeline presented in this paper. We can describe the whole HDR reconstruction process with a single PFA camera through the following main blocks:

1. The *recovery network* is in charge of synthesizing the response of a regular PFA camera (in terms of AoLP and DoLP) from an image taken with an additional external linear polarizer. The network architecture includes some components, referred to as *mosaiced convolutions* from the PFA demosaicing network (PFADN) [72]; these components have been shown to be effective for demosaicing the PFA raw camera images to obtain a full-resolution intensity image and AoLP.
2. The image acquired via the PFA camera with the additional filter is merged with the recovery network output using the camera model presented in [15] to produce an HDR image. Note that this step is deterministic and not learning-based since it is a direct application of the theory presented in the previous section.
3. Finally, the *tone mapping network* takes as input the computed HDR image and is designed to make the output perceptually similar to the ground truth.

In the following part of the current section, we will describe in detail the mentioned components, namely, the recovery network, HDR generation, and tone mapping network.

### 4.1. Recovery Network

As already discussed, we acquire the scene with a single PFA camera (denoted as $cam_{f}$) with an external linear polarizer mounted with an angle $\alpha_{f}$. As a result, the camera captures a mosaiced image $I_{f}$, where the four channels (associated with each internal filter) are interleaved. Suppose that $\theta_{f}$ and $D_{f}$ are the angle and degree of polarization of the acquired data and let θ and D be the actual AoLP and DoLP of the incoming light, which would have been captured by the camera without the additional filter. The recovery network (RecNet) takes as input the mosaiced image $I_{f}$ and aims at recovering AoLP (θ) and DoLP (D), which is essentially equivalent to the removal of the additional polarizer filter.

**Figure 4:**
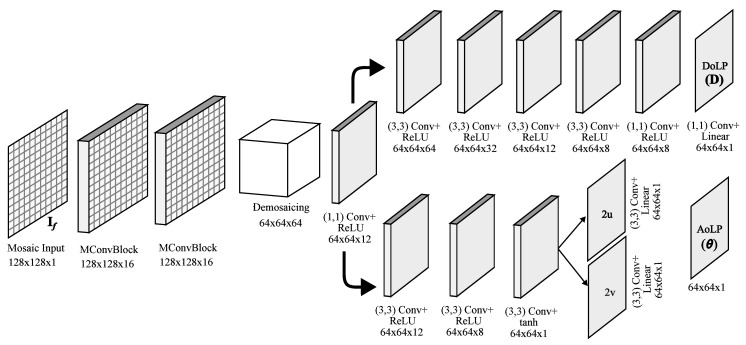
RecNet architecture: the input is a 128×128 mosaiced image taken by a PFA camera with an external filter. Two subsequent mosaic convolutions extract a feature map of size 128×128×16, which is demosaiced into a 64×64×64 cube. The network then computes the degree of linear polarization D and angle of polarization θ in parallel.

RecNet is built using mosaiced convolution (MConv) operations. These convolutions are designed to extract features from a polarimetric mosaic input by taking into account the PFA pattern; details of MConv can be found in [72]. Figure 4 shows the complete architecture of the proposed network. It takes a tensor of size w×h×1 as the input, and then two MConv blocks extract features from $I_{f}$, giving a feature map of size w×h×16, where 16 is the total number of computed filters (or depth). In the following layer, such a tensor is demosaiced into a (w/2)×(h/2)×64 tensor, concatenating the four channels depthwise. After performing a 1×1 2D convolution followed by ReLU activation, we obtain a (w/2)×(h/2)×12 tensor. After that, the network splits into two parts: the first branch of the network is responsible for recovering the DoLP D of a scene, while the second branch estimates the AoLP θ.

Regarding the first part, we know that D∈[0,1]; therefore, it can be considered a grayscale image normalized between 0 and 1. To produce D, 2D convolutions with ReLU activations are applied in each layer to produce a sequence of 64, 32, 12, 8, 8, 1 features maps. After the last convolution, the network produces an (w/2)×(h/2) intensity image that represents the predicted DoLP of the scene without the filter.

The second network branch aims at producing the scene’s original AoLP θ. In order to do that, it performs three 2D convolutions, obtaining tensors with depths of 12,8,1. Unlike [72], we are not interested in the full-resolution output, so we do not perform upscaling, and directly output the two vector components (2u,2v) of the AoLP θ of the scene, such that:

(4) $$\theta =\frac{1}{2}\mathrm{atan}2\left(2v,2u\right).$$

The loss function employed for RecNet is as follows:

(5) $$L_{RecNet}\left(D,\hat{D},A,\hat{A}\right)=\gamma L_{D}\left(D,\hat{D}\right)+\left(1-\gamma \right)L_{A}\left(A,\hat{A}\right)$$

where we set γ=0.5, while $L_{D}$ and $L_{A}$ are, respectively, the loss functions for the degree and the angle:

(6) $$\begin{array}{ccc} L_{D}\left(D,\hat{D}\right) & = & \left(1-\beta \right)|D-\hat{D}|+\beta (1-\mathrm{SSIM}(D,\hat{D}))\\ L_{A}\left(A,\hat{A}\right) & = & ∥A-\hat{A}{∥}_{2}^{2} \end{array}$$

where SSIM is the structural similarity function [73] between the predicted and ground truth DoLP, while $\hat{A}$ is the two-channel image (2u,2v) and A=(cos(2θ),sin(2θ)). The weighting parameter β in our settings has been set to 0.85.

### 4.2. HDR Generation

The four intensity values $I_{0},I_{45},I_{90},I_{135}$ captured by $cam_{f}$ with the exposure time $t_{c}$ are related to the actual irradiance ${\hat{I}}_{i}$ (defined in Equation (3)), as follows:

(7) $$g\left(I_{i}\right)={\hat{I}}_{i}t_{c}$$

where g(·) is the inverse camera response function (ICRF). Then, expanding ${\hat{I}}_{i}$ as in Equation (3), we can easily substitute ${\hat{I}}_{i}t_{c}$ with $It_{i}$, where

(8) $$t_{i}=\frac{1}{4}t_{c}\left(1+D\cos(2\theta -2\alpha_{f})\right)\left(1+D_{f}\cos\left(2\theta_{f}-2\alpha_{i}\right)\right).$$

Indeed, we can define the $t_{i}$ (with $i\in \{0^{\circ },45^{\circ },90^{\circ },135^{\circ }\}$) as *equivalent exposure times*. As a consequence, the acquired images $I_{0},I_{45},I_{90},I_{135}$ can be seen as the intensities one would have observed by exposing the image, respectively, with times $t_{0},t_{45},t_{90},t_{135}$, without the polarizers. Since each pixel is an independent observation, the equivalent exposure times vary across the image and, thus, are considered per-pixel.

Let $t_{c}$ be the shutter speed of $cam_{f}$. After RecNet produces the scene’s AoLP θ and DoLP D, these are combined with the angle $\theta_{f}$ and degree $D_{f}$ (i.e., observed by $cam_{f}$) to compute the equivalent exposure time as in Equation (8). Finally, the intensities from the four filters $I_{0},I_{45},I_{90},I_{135}$, along with the equivalent exposure times $t_{i}$, are used to compute HDR values via the following weighted average:

(9) $$I_{HDR}=\frac{\sum_{i\in T}w\left(I_{i}\right)\frac{I_{i}}{t_{i}}}{\sum_{i\in T}w\left(I_{i}\right)}$$

where T={0,45,90,135} is the set of filter orientations and *w* is the Gaussian weight function, defined as $w\left(x\right)=\exp(-\frac{{\left(x-0.5\right)}^{2}}{2\sigma^{2}})$. The idea behind *w* is to give more importance to values that are closer to the middle of the response and, consequently, assign a higher weight to the properly exposed pixels. By computing the value $I_{HDR}$ for each observed pixel, we obtain the complete HDR image H.

### 4.3. Tone Mapping Network

**Figure 5:**
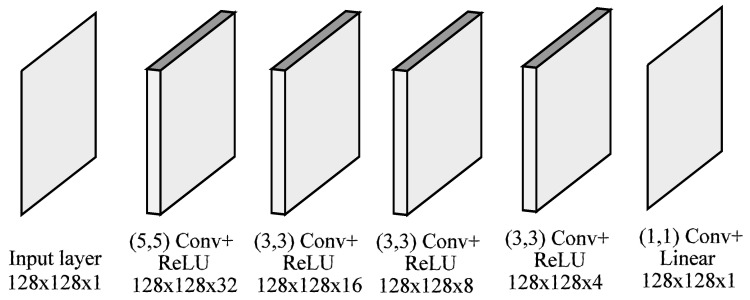
Improvement network: The HDR images generated using the technique described in Section 4.2 were first converted into the LDR domain via tone mapping. The tone-mapped images were then used as input to the network, where a sequence of 2D convolutional layers extracted features and mapped them to the final output.

The tone mapping network (TMNet) takes H as the input and performs a tone mapping operation, producing the final image that can be visualized. As shown in Figure 5, the network consists of five 2D convolutions layers, which extract 64, 32, 16, 8, 1 feature maps, respectively; the last one corresponds to the tone-mapped HDR image $\hat{H}$. In each layer, the receptive field is progressively decreased, having kernels of sizes 9, 5, 3, 1, 1, respectively. This results in mapping only important features to the output layer. The ReLU function is used to activate all layers, except the output. The loss function of TMNet is designed to take into account the overall quality of the output image; for this reason, we opted for a linear combination of the SSIM and a perceptual loss:

$$L_{TMNet}\left(\bar{H},\hat{H}\right)=\alpha \left(1-\mathrm{SSIM}(\bar{H},\hat{H})\right)+\left(1-\alpha \right)L_{per}\left(\bar{H},\hat{H}\right)$$

where $\bar{H}$ is the ground truth HDR image and α is a balancing value that we empirically set to 0.5 after some preliminary tests. The function $L_{per}$ is a perceptual loss function that takes as input the two HDR images, $\bar{H}$ and $\hat{H}$, and computes the sum of the squared differences of the output of the intermediate feature maps $\phi_{l}$ from the predefined layers of the VGG-19 architecture [74]. In particular:

(10) $$L_{per}\left(\bar{H},\hat{H}\right)=\sum_{l\in L}{\left(\phi_{l}\left(\bar{H}\right)-\phi_{l}\left(\hat{H}\right)\right)}^{2}$$

where L={pool1,pool2,pool3} are the VGG layer identifiers.

## 5. Experimental Section

In this section, we first describe the process we employed to acquire a suitable dataset that was used to train both RecNet and TMNet, along with the training process. Then, we evaluated the quality of the proposed method by comparing it with state-of-the-art methods, both quantitatively and qualitatively.

### 5.1. Dataset Acquisition

We used a single PFA camera setup to acquire the data needed to train the model and assess the quality of the final HDR reconstruction. We employed a FLIR Blackfly monochrome PFA camera with the Sony IMX250MZR sensor (Tokyo, Japan), which gave raw images that were 2448×2048 pixels (before demosaicing). The camera ICRF was calibrated using [75], and applied to all of the input images as a preprocessing step.

We positioned the camera on a stable support for each unique scene. First, we captured a single picture with the additional external filter. Following that, we captured a sequence of 30 images without the filter, gradually increasing the exposure time. The whole acquisition process lasted a couple of seconds, and we captured the static scenes in order to be able to perform reasonable comparisons with other methods. For each set of images, we demosaiced all 30 exposures to obtain the scene intensities, which were used to compute the HDR ground truth for the observed scenes using the method described in [16]. In total, 20 high-dynamic indoor and outdoor scenes with different ambient conditions were captured. Finally, the acquired images were divided into blocks of size 128×128, resulting in a total of 5100 extracted patches.

### 5.2. Training Procedure

Our HDR reconstruction pipeline was composed of two independent networks, which were trained using different portions of datasets. We recall that the objective of the recovery network is to take as input a mosaiced image from a PFA camera with an external linear polarizer and estimate the original scene features in terms of AoLP and DoLP. In order to do this, we specifically designed a dataset to simulate the outcome of such acquisitions in real-world settings. Indeed, given a raw mosaiced image taken with a PFA camera, it is quite straightforward to simulate the effect of an additional filter outside the lenses. Recalling Equation (3), the observed intensity is connected to the other parameters as follows:

(11) $${\hat{I}}_{i}=\frac{1}{4}S_{0}\left(1+D\cos(2\theta -2\alpha_{f})\right)\left(1+D_{f}\cos\left(2\theta_{f}-2\alpha_{i}\right)\right)$$

where $S_{0}$ is the intensity captured by the PFA camera (the first Stokes parameter), D and θ are the actual DoLP and AoLP of the scene, and $\alpha_{i}$ is the angle of the internal polarizer filter array $\alpha_{i}\in \left\{0^{\circ },45^{\circ },90^{\circ },135^{\circ }\right\}$. The values $D_{f}$ and $\theta_{f}$ depend on the external filter and can be easily generated to simulate different acquisition conditions to simulate a high number of training images without physically adding and removing the filter. Therefore, since RecNet requires several images for the training process, we synthetically generated filtered images starting from real PFA camera data. Specifically, we used 50% of our data and added the dataset presented in [76] (“*sunny outdoor*” acquisitions). The ground truth AoLP and DoLP are directly calculated from the captured dataset, while the network input is synthesized by applying Equation (11). We simulated random $D_{f}$ between 0.8 and 1 and randomized the value $\theta_{f}$ to avoid completely dark images in individual channels.

The tone mapping network was trained exclusively on our dataset, with the HDR ground truth computed from exposure bracketing, as previously explained; tone mapping was applied as described in [77]. We chose random exposures from each scene, avoiding complete dark or white images so that the presented model could learn to reconstruct the HDR irrespective of the exposure time.

For both networks, datasets were divided into 70/30 for training and testing, respectively, and the Adam optimizer was used with a $10^{-3}$ learning rate and trained up to convergence (∼30 epochs for each network). The model was implemented using TensorFlow and NumPy libraries and the process ran on an NVIDIA GeForce RTX 2080 Ti GPU with a batch size set to 64.

### 5.3. Quantitative Analysis

In order to evaluate the performance of the proposed technique, we designed a set of experiments to first assess the effectiveness of RecNet and then to test the final HDR reconstruction by comparing the outcomes of several approaches.

**Figure 6:**
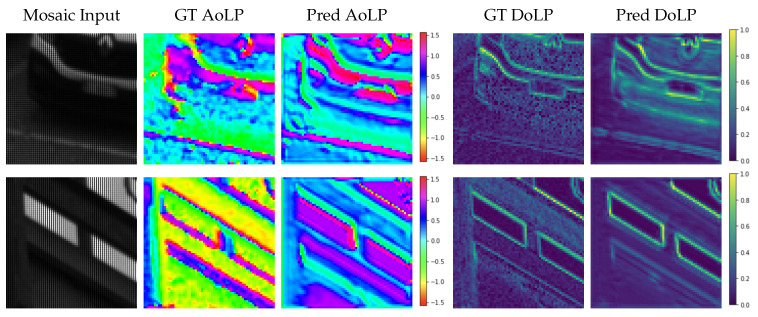
Outputs from RecNet. From left to right: input image with the synthesized external filter, ground truth AoLP, predicted AoLP (both in radians), ground truth DoLP, predicted DoLP. In the first row, we can see good reconstruction in terms of both the angle and degree of polarization. In the second row, the angle is not always correct, but the predicted degree is good. This does not affect the HDR outcome since, for high degrees, the corresponding angle is correct, and for lower degrees (the majority of this scene), the original angle is actually discarded.

As already discussed, we trained RecNet with synthesized filters, starting from our dataset and other datasets. Figure 6 shows two example outputs of RecNet from the test set; the input is the mosaiced image with the (simulated) external filter. The degree of linear polarization was successfully recovered by the network; the angle results were almost good in the first row, while in the second, we can see some differences. This is actually not a problem for our application because we are interested in recovering the original scene angles for regions with high DoLP, where the angle is relevant. Indeed, in regions where the DoLP is very close to zero, the camera model described by Equation (8) discards the angle value since the term has a minimal effect. As a consequence, we aim to recover only the angles for which the degree is quite high. The overall average angle loss for the test set was $36^{\circ }$.

The performance of the presented method was verified by comparing it to some existing single-image-based HDRI techniques. In total, seven state-of-the-art methods were selected for comparison; among these, five were learning-based and two were algorithmic approaches. In particular, KO [48] is an inverse tone mapping technique while Wu et al. [3] presented an HDR technique specifically designed for PFA cameras. The learning-based HDRI methods we compared with are the two-stage HDR [78], HDRCNN [49], Deep-HDR [79], ExpandNet [11], and DPHDR [13]. For all of the listed methods, we used the implementations of the authors as well as pre-trained weights where possible. Note that Wu et al. [3] and DPHDR [13] are methods specifically designed to generate HDR images by taking a PFA image as input, the rest of the techniques are not designed for PFA cameras; hence, intensity images are provided as input. We used our acquired dataset for a comparison on the test set, and Reinhard tone mapping [80] was applied to the HDR outputs only for methods that do not directly output a tone-mapped image. In all of the experiments, the ground truth was computed as previously described, and the output of our proposed method was generated by using a single image captured with an additional linear polarizer. Moreover, in order to show the effectiveness of the tone mapping network proposed in our approach, we also compared the results obtained from our model without the TMNet. In other words, we directly tone-mapped the HDR output with [80], excluding the second network.

Table 1 shows the comparison results. For all techniques, we computed the peak signal-to-noise ratio (PSNR) and multi-scale structural similarity (MS-SSIM) [81]. PSNR is computed as follows:

(12) $$\mathrm{PSNR}\left(X,X_{GT}\right)=20\;log_{10}\frac{MAX_{I}}{\sqrt{\mathrm{MSE}(X,X_{GT})}}$$

where $MAX_{I}$ is the maximum possible value among image pixels, and MSE denotes the mean squared error between the ground truth $X_{GT}$ and the predicted image *X*. MS-SSIM is an extension of the SSIM [73] function, which calculates and combines the SSIM of an image at different scales. Given a pair of ground truth $X_{GT}$ and predicted images *X*, MS-SSIM is computed as follows:

(13) $$\mathrm{MS}\text{-}\mathrm{SSIM}\left(X,X_{GT}\right)={\left[l_{M}\left(X,X_{GT}\right)\right]}^{\alpha_{M}}.\prod_{j=1}^{M}{\left[c_{j}\left(X,X_{GT}\right)\right]}^{\beta_{j}}{\left[s_{j}\left(X,X_{GT}\right)\right]}^{\gamma_{j}}$$

where *M* is the number of scales, $l_{j},c_{j}$, and $s_{j}$ refer to the luminance, contrast, and similarity measures of the *j*-th scale, respectively. Parameters $\alpha_{j},\beta_{j}$, and $\gamma_{j}$ are used to assign weights to different measurements for the *j*-th scale.

We can see that our method produced the highest average PSNR (dB) and MS-SSIM for the test dataset. Although Wu et al. [3] and DPHDR are built for PFA cameras, they do not provide any significant improvements, for two reasons. First: The DoLP of the scenes is generally very small; hence, it fails to take full advantage of polarimetric data. Second: DPHDR is a learning-based method but the model provided by the authors is not suitable to work with such data or it overfits some patterns and does not generalize. Moreover, DPHDR works on patches of size 512×512 and does not provide a way to connect these patches to produce a full-resolution output of (1024×1224). We also notice that our method without TMNet (second row) still offers results that are better when compared to other methods, meaning that the output provided by RecNet is effective in simulating the second camera with the additional filter. The rest of the data-driven techniques (based on a single image) presented in the table offer lower outcomes: the best is the two-stage HDR (19.59 dB), which can be compared to our model without TMNet (20.51 dB).

### 5.4. Qualitative Analysis

**Figure 7:**
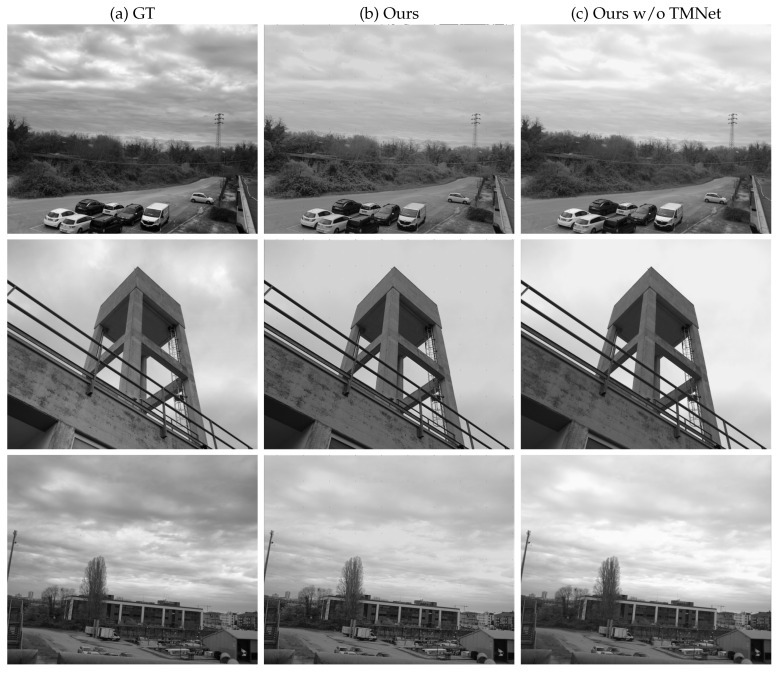
Qualitative comparison from our test set with and without TMNet. From left to right: ground truth HDR image, output of our proposed complete pipeline, tone-mapped output without applying our TMNet.

**Figure 8:**
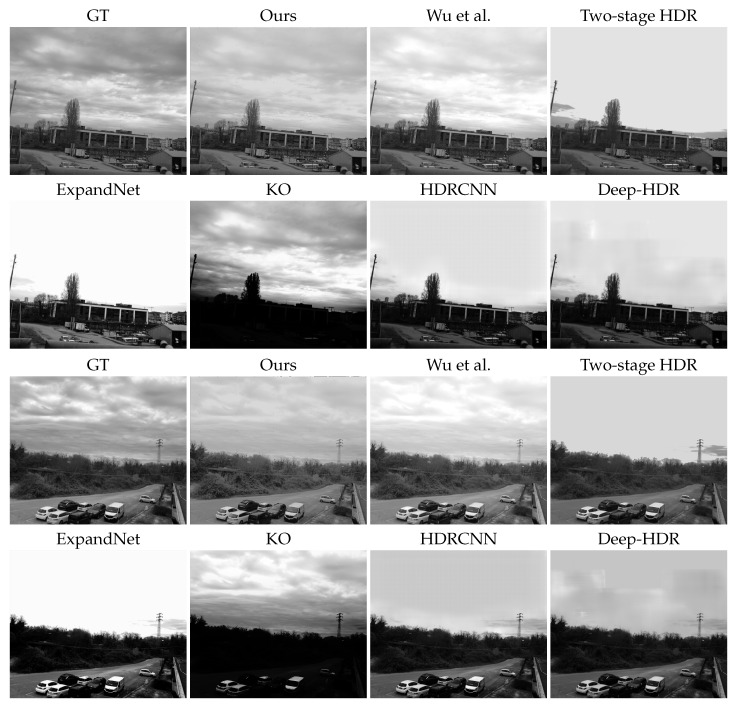
Qualitative results for all compared techniques against ground truth from our test data (with synthetic filter). Wu et al. refers to [3].

As the last part of our analysis, we qualitatively compared some of the results from our method with respect to other approaches. The first thing that we wanted to compare is the final result with or without the contribution of the tone mapping network. Figure 7 shows three example images that were obtained by applying the full proposed pipeline with TMNet (second column) and directly applying the tone mapping [80] after HDR generation (third column). We can notice that excluding TMNet leads to the worst results and saturated regions, especially in the sky area, whereas our full approach manages to recover better details in such challenging settings. Figure 8 compares the quality of all of the proposed methods from a scene with a simulated additional filter. Our method demonstrates superior quality results compared to other methods, even though we employed a synthetic filter approach to train RecNet. This shows that the proposed training procedure is indeed effective in simulating real-environment conditions when employing the external filter in our method.

**Figure 9:**
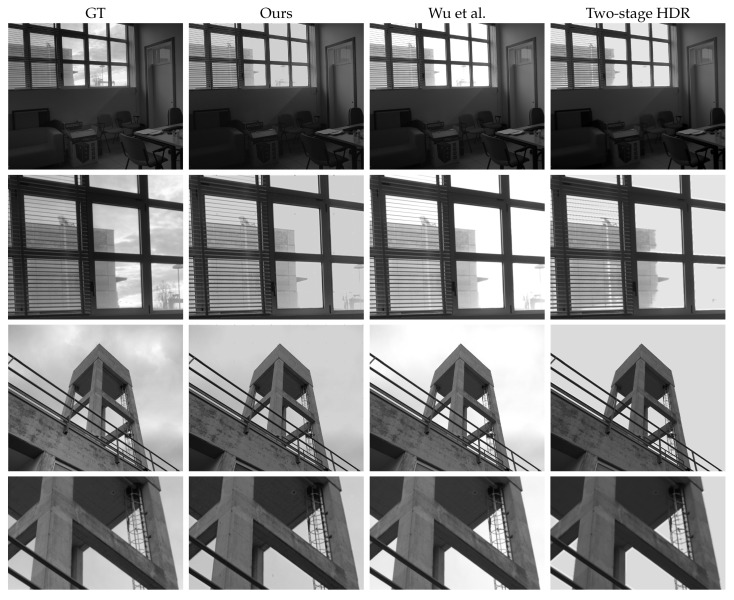
Best HDR methods with details. From left to right: ground truth (GT, produced using classical multi-exposure techniques), our proposed method, the PFA-based method Wu et al. [3], and learning-based single image HDRI Two-stage HDR, respectively. For better visualization, zoomed-in patches of rows 1 and 3 are shown in rows 2 and 4, respectively.

**Figure 10:**
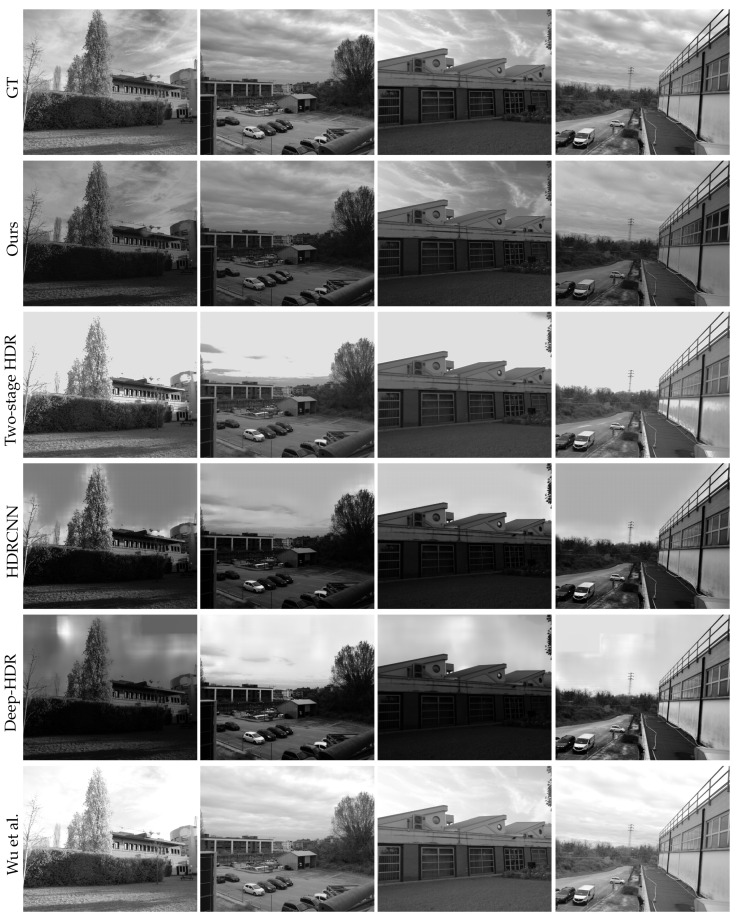

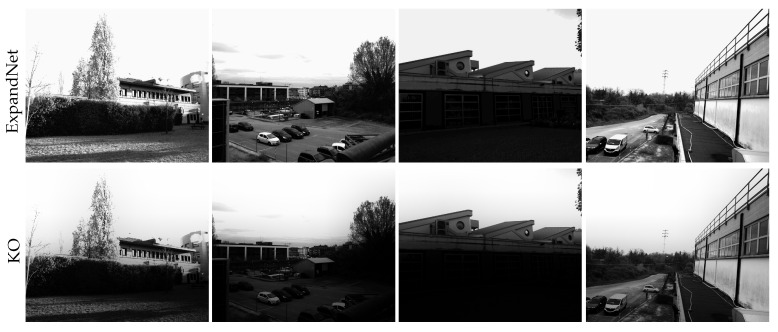
Qualitative comparison of the test set captured with a real filter outside the PFA camera lenses.

Finally, Figure 9 and Figure 10 are produced with our acquired dataset and physical filter mounted on the camera. In particular, Figure 10 shows a qualitative comparison of all the methods, and Figure 9 shows a close-up of the three best methods, according to Table 1. In detail, the third column in Figure 9 shows the PFA-based HDRI Wu et al. [3] and the last one is the single image HDR reconstruction method, denoted as the *two-stage HDR* method. In general, we notice that Wu et al. failed to deal with the oversaturated regions of the image, resulting in overall brighter results. Moreover, in Figure 10, the results generated by [3] have overexposed regions; this works better when compared to other techniques except ours. The technique proposed by [3] has been specifically designed for PFA cameras, but due to the lower DoLP of the scenes, the four filter images taken by PFA cameras have, approximately, the same exposure times and, consequently, there is not enough increase in the dynamic range of the resulting HDR image. On the other hand, the output of the two-stage HDR in the overexposed regions is somehow similar to ground truth, but it could not recover low-level details in complicated areas. Our method produced better quality HDR images, which exhibited a contrast similar to the ground truth while retaining fine details, which is evident in the zoomed-in patches.

Qualitative results of other competing techniques can be found in Figure 10. The techniques have their own shortcomings and they all suffer from different kinds of artifacts, leading to poor results. The under-performance of deep learning-based techniques, such as ExpandNet, HDRCNN, and Deep-HDR, can be explained by different reasons. One possible reason is the training process, as these methods are trained on specific datasets, and due to overfitting, it is difficult for these pre-trained networks to reproduce better-quality results. Another reason could be the lack of a standard protocol for HDR reconstruction problems, such as dataset and evaluation conditions. These methods might report good performances with their set conditions but they fail when dealing with different scenes.

## 6. Conclusions

In this paper, we proposed an HDR reconstruction method based on a data-driven virtual stereo PFA camera setup, requiring only a single shot with an additional external linear polarizer. The idea is based on a previous method that uses a physical stereo setup with two synchronized PFA cameras to generate HDR images that exploit linear polarizers. The virtual setup proposed in this work overcomes the drawbacks of the previous approach by discarding one camera while taking advantage of the polarimetric imaging model. To do so, we introduced a novel CNN architecture, denoted as the recovery network (RecNet), which is designed to eliminate the need for a second camera. Indeed, such a model is trained to predict the original angle and degree of polarization from the raw mosaiced image taken with the additional filter. In this way, the recovery network output is directly used to compute the HDR image. Finally, the HDR image is further improved through a second CNN model that performs optimal tone mapping on the input data. In addition, since there are no publicly available datasets for this kind of task, we created a new dataset that is designed for learning-based polarimetric HDR imaging. We carefully performed the data acquisition procedure so that we could effectively acquire challenging scenes, and at the same time, we compared the results obtained with our method and other methods designed for single-shot HDRI. Experiments show that the described two-stage neural network pipeline increases the range of estimated exposure times and improves the final image quality even further when applying the second network. As part of future work, we will aim to improve the two CNN model performances in terms of both the angle recovery and tone mapping, so that the proposed system can be applied in more challenging environments.

## Figures and Tables

**Table 1:** Comparison between HDR methods with Reinhard tone mapping [80] applied. Bold font indicates the best value in each column.

Method	PSNR (dB)	MS-SSIM
Ours	**23.0670 ± 2.1362**	**0.9756 ± 0.0145**
Ours w/o TMnet	20.5114 ± 3.7150	0.9741 ± 0.0121
Wu et al. [3]	18.9489 ± 4.1196	0.9706 ± 0.0188
KO [48]	12.9069 ± 1.6990	0.5067 ± 0.1880
DPHDR [13]	12.5211 ± 2.4826	0.6465 ± 0.0786
Two-stage HDR [78]	19.5877 ± 3.1949	0.9658 ± 0.0230
Deep-HDR [79]	16.6983 ± 1.8659	0.7840 ± 0.1401
ExpandNet [11]	14.6473 ± 2.5156	0.8113 ± 0.0788
HDRCNN [49]	14.8472 ± 2.8604	0.7202 ± 0.1661

## Data Availability

The data presented in this study are available on request from the corresponding author.
